# Sensitivity of Acute Myelocytic Leukemia Cells to the Dienone Compound VLX1570 Is Associated with Inhibition of the Ubiquitin-Proteasome System

**DOI:** 10.3390/biom11091339

**Published:** 2021-09-10

**Authors:** Karthik Selvaraju, Kourosh Lotfi, Johannes Gubat, Maria Miquel, Amanda Nilsson, Julia Hill, Lasse D. Jensen, Stig Linder, Pádraig D’Arcy

**Affiliations:** 1 Department of Biomedical and Clinical Sciences (BKV), Linköping University, SE-581 85 Linköping, Sweden; karthik.selvaraju@liu.se (K.S.); kourosh.lotfi@liu.se (K.L.); johannes.gubat@liu.se (J.G.); mariamiquel095@gmail.com (M.M.); amani157@student.liu.se (A.N.); julhi958@student.liu.se (J.H.); stig.linder@liu.se (S.L.); 2 Department of Hematology, Linköping University Hospital, SE-581 85 Linköping, Sweden; 3 Department of Health, Medical and Caring Sciences (HMV), Linköping University, SE-581 85 Linköping, Sweden; lasse.jensen@liu.se; 4 Department of Oncology-Pathology, Karolinska Institute, SE-171 76 Stockholm, Sweden

**Keywords:** acute myeloid leukemia, proteasome, ubiquitin, deubiquitinase, zebrafish

## Abstract

Dienone compounds with a 1,5-diaryl-3-oxo-1,4-pentadienyl pharmacophore have been widely reported to show tumor cell selectivity. These compounds target the ubiquitin-proteasome system (UPS), known to be essential for the viability of tumor cells. The induction of oxidative stress, depletion of glutathione, and induction of high-molecular-weight (HMW) complexes have also been reported. We here examined the response of acute myeloid leukemia (AML) cells to the dienone compound VLX1570. AML cells have relatively high protein turnover rates and have also been reported to be sensitive to depletion of reduced glutathione. We found AML cells of diverse cytogenetic backgrounds to be sensitive to VLX1570, with drug exposure resulting in an accumulation of ubiquitin complexes, induction of ER stress, and the loss of cell viability in a dose-dependent manner. Caspase activation was observed but was not required for the loss of cell viability. Glutathione depletion was also observed but did not correlate to VLX1570 sensitivity. Formation of HMW complexes occurred at higher concentrations of VLX1570 than those required for the loss of cell viability and was not enhanced by glutathione depletion. To study the effect of VLX1570 we developed a zebrafish PDX model of AML and confirmed antigrowth activity in vivo. Our results show that VLX1570 induces UPS inhibition in AML cells and encourage further work in developing compounds useful for cancer therapeutics.

## 1. Introduction

**Figure 1:**
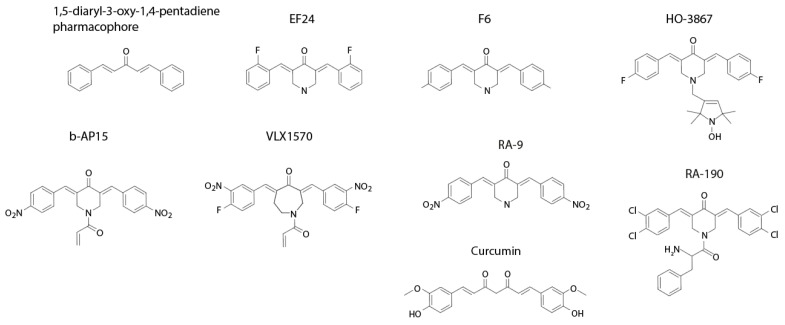
Structures of compounds discussed in the text.

Compounds with a 1,5-diaryl-3-oxo-1,4-pentadienyl pharmacophore (Figure 1) (hereby referred to as “dienones”) have attracted considerable interest as anticancer agents [1]. Although the presence of α,β-unsaturated ketones that function as Michael acceptors raises the possibility of general cysteine reactivity, many compounds of this class have been documented as tumor selective [1,2,3]. A number of groups have shown that dienone compounds affect the ubiquitin-proteasome system (UPS) [2,4,5,6]. Malignant cells are sensitive to disturbances in the UPS primarily due to increased levels of protein production and a dependency on UPS-mediated degradation for the maintenance of homeostasis [7]. Several classes of dienone compounds have been shown to induce redox stress, either directly by inhibiting scavenging pathways or via the acute induction of reactive oxygen species (ROS) due to defective protein folding and degradation. Glutathione depletion caused by Michael-acceptor-containing compounds may be related to the formation of high-molecular-weight protein complexes that trigger cytotoxic responses [8,9].

Acute myeloid leukemia (AML) is a hematologic malignancy characterized by the accumulation of immature myeloid cells in the blood and bone marrow [10]. The neoplastic cells from AML patients are cytogenetically diverse, with approximately 10% of AML patients displaying a complex karyotype associated with multiple chromosome rearrangements, a high relapse rate, and a poor prognosis [11,12]. The spectrum of abnormalities of those with complex karyotypes includes a predominance of chromosomal imbalances including the losses of chromosome regions at 5q, 17p, and 7q, and gains at 8q, 11q, and 21q. The prominent features of complex karyotype cases are the frequent loss of 17p, a high prevalence of DNA amplifications, and/or mutation in the *TP53* gene. Although only present in a subset of AML cases, the mutational status of *TP53* is a critical prognostic indicator associated with very poor outcomes [13].

Dienone compounds generally show limited solubility. Both b-AP15 and VLX1570 (Figure 1) have been used in formulations containing Chremophor EL. Despite passing Good Laboratory Practice (GLP) toxicity testing, VLX1570 toxicity was encountered in a Phase 1 trial [14]. Toxicity was subsequently also observed with bortezomib using the same formulation [14]. PEGylation may be one strategy to approach this problem [15]. Despite problems related to poor solubility, these compounds show promising antineoplastic activities in a number of animals models, and the development of more soluble compounds is ongoing.

It was recently reported that AML cell lines show a high degree of sensitivity to VLX1570 [16]. Sensitivity was reported to be due to the generation of ROS and induction of ER stress. Although these results are promising, considering the severity of the disease, it cannot be excluded that the general reactivity of the VLX1570 molecule contributes to cytotoxicity [9]. AML cells have been reported to be sensitive to the depletion of reduced glutathione by electrophiles [17]. We here studied the effects of the dienone VLX1570 on the viability of AML cells, both cell lines and patient-derived primary cells. We were interested in exploring how antiproliferative responses correlated with UPS inhibition, reduction in the cellular levels of reduced glutathione, and the generation of protein aggregates. Our results support the view that VLX1570 induces proteotoxic stress, ER stress, and apoptosis. Apoptosis does not, however, appear to be required for the cytotoxicity of VLX1570 pointing to a robust mechanism of cell death induction.

## 2. Materials and Methods

### 2.1. Cell Lines

MOLM-14 (ACC 777), KG-1a (ACC 421), Kasmui-1 (ACC 220), and HNT-34 (ACC 600) were purchased from Leibniz-Institute DSMZ German Collection of Microorganisms and Cell Cultures GmbH (Brunswick, Germany). AML cells were cultured in RPMI-1640 medium supplemented with 10% fetal bovine serum and 1% penicillin/streptomycin. For the isolation of patient-derived AML cells, whole blood was fractionated using a standard Ficoll gradient protocol. AML blasts were removed for culture in HSC media (Lonza, Basel, Switzerland) supplemented with IL3, IL6, TPO, FLT3 (10 ng/mL), and SCF (25 ng/mL). Mutation analysis was performed by MLL (Munich Leukemia Laboratory). The use of patient-derived cells in this study was approved by the regional ethics committee in Linköping, Sweden (DNR 2017/384-31).

### 2.2. Reagents and Antibodies

VLX1570 was synthesized by OnTarget Chemistry AB (Uppsala, Sweden). BSO, Thapsigargin, Z-VAD-FMK were obtained from Sigma Aldrich (Darmstadt, Germany). Antibodies were from the following sources: ubiquitin Lys48 (Merck, Kenilworth, NJ, USA), active caspase-3, BIP, and XBP1S obtained from Cell Signaling Technology (Danvers, MA, USA). PARP and antiheme oxygenase-1 were obtained from BD Biosciences (San Jose, CA, USA). β-actin and HSP70B were obtained from Sigma-Aldrich (Darmstadt, Germany). USP14 was obtained from Bethyl Laboratories (Montgomery, TX, USA).

### 2.3. Cell Viability Assays

AML cells were treated with indicated compounds or DMSO as a reagent control. Viability was determined at indicated time intervals following drug exposure using either MTT assay (Promega, Madison, WI, USA) or AO/PI staining and analysis with a benchtop cytometer, (Cellometer^®^ K2, Nexcelom Bioscience, Lawrence, MA, USA). Data obtained was used to estimate IC_50_ values using GraphPad Prism software (San Diego, CA, USA).

### 2.4. SDS PAGE and Immunoblotting

Cell-extract proteins were resolved by Tris-Acetate PAGE and Bis-Tris gels (Invitrogen, Carlsbad, CA, USA) and transferred onto polyvinylidene difluoride membranes (PVDF), blocked in 5% milk in PBST, and incubated overnight with indicated antibodies. Membranes were washed and incubated with HRP-conjugated antirabbit IgG or antimouse IgG (Cell Signaling Technology, Danvers, MA, USA), followed by detection using BioRad Gel Doc. For Coomassie staining, proteins were resolved on PAGE gels (Invitrogen, Carlsbad, CA, USA) followed fixation and staining with Bio-Safe Coomassie G-250 stain (Bio-Rad Laboratories, Hercules, CA, USA) according to manufacturer’s instructions.

### 2.5. Glutathione Analysis

For assessment of glutathione (GSH) levels, cells were seeded at a concentration of 5000 cells/well in opaque 96-well culture plates (Nunc, Roskilde, Denmark) and treated as indicated. Cells were treated with the indicated concentration range of VLX1570 for indicated time points, followed by immediate removal of culture medium. GSH levels were measured using GSH-Glo kit (Promega, Madison, WI, USA) according to the manufacturer’s instructions. Briefly, cells were lysed for 30 min in buffer containing luciferin-NT and glutathione S-transferase (GST), at concentrations where GST effectively catalyzes the generation of luciferin from luciferin-NT proportional to the concentration of GSH in the sample. Luciferin was then detected as a luminescent signal by incubation in luciferin detection reagent containing luciferase for 15 min. Luminescence was measured using the GloMax1 explorer microplate reader (Promega, Madison, WI, USA).

### 2.6. Developmental Toxicity Assay

At indicated time postfertilization, 20 zebrafish embryos were incubated in E3 embryo medium containing 0.003% 1-phenyl-2-thiourea (PTU) and various concentrations of the tested drugs. The embryos were kept at 28.5 °C and analyzed for survival and gross developmental phenotypes under a stereomicroscope.

### 2.7. Zebrafish Tumor Model

AML cells were labeled with 1,10-dioctadecyl-3,3,303′-tetramethylindocarbocyanine (DiI), as previously described [18]. In brief, 70–80% confluent AML cells were washed with DPBS and incubated with DiI at a final concentration of 4 µg/mL for 30 min at 37 °C. After labeling, cells were washed twice in DPBS and kept on ice prior to implantation in zebrafish embryos. Transgenic Tg(fli1:EGFP)y1 zebrafish embryos [19] were raised in E3-medium supplemented with PTU. AML cells were resuspended at approximately 10^8^ cells per mL in cell growth medium, and approximately 400 cells in a 4 nL volume were implanted in the perivitelline space via sharp glass needles (world precision instruments, pulled in a PC-10 needle puller, Narishige, Tokyo, Japan) using a microinjection setup (MINJ-D, TriTech Research, Los Angeles, CA USA). Following injection, embryos were sorted for specific implantation of tumor cells in the perivitelline space and absence of cells in circulation under a fluorescent microscope (SMZ1500, Nikon, Tokyo, Japan) and placed in E3 embryo medium containing 0.2 mM PTU. Three days following tumor implantation, the embryos were anesthetized in MS-222 (0.04%, Sigma-Aldrich, St. Louis, MO, USA). Primary tumor sizes as well as the extent of local, peripheral, and hematogenous dissemination/metastasis of tumor cells were visualized under the fluorescent microscope. Results are shown as the average ± standard error of the mean of tumor volumes or number of cells present posterior to the anal opening. All animal experiments were approved by Linköpings Djurförsöksetiska Nämnd (N89/15).

### 2.8. CMap Analysis

The Broad Institute CMap database was accessed through https://clue.io/touchstone (accessed on 7 June 2021). The analysis resulted in lists of genes, the knockdown of which resulted in gene expression profiles that are similar to those induced by the compounds/drugs of interest. Ranking was determined through *tau*-scores (see, https://clue.io/connectopedia/connectivity_scores, accessed on 7 June 2021). *Tau*-scores of +90 or higher are considered to be strong scores.

## 3. Results

### 3.1. Properties of Dienone Compounds

Dienone compounds with the 1,5-diaryl-3-oxo-1,4-pentadienyl pharmacophore (Figure 1) are occasionally referred to as curcumin analogues/curcuminoids [20]. For the last >20 years, the laboratory of Jonathan Dimmock has described a large number of 1,5-diaryl-3-oxo-1,4-pentadienyl compounds and showed them to be strong inducers of tumor cell apoptosis, demonstrating a preferential activity on tumor cells over normal cells [3]. The compound EF24 (NSC716993, Figure 1) was shown to induce cell cycle arrest, apoptosis of cancer cells [21], and antineoplastic effects in animal models [22,23]. The properties of some compounds in this series are displayed in Table 1. 1,5-diaryl-3-oxo-1,4-pentadienyl compounds contain multiple Michael acceptors, leading to an expected thiol reactivity. These compounds are flagged as PAINS (pan-assay interference compounds). They show general low solubility (Table 1) and often require formulations containing detergents and castor oil for in vivo injections. Solubility (ESOL) [24] and consensus LogP were calculated using SwissADME [25]. References with regard to targets are provided in the text.

We used the Broad Institute Library of Integrated Network-Based Cellular Signatures (LINCS) Center for Transcriptomics resource to compare gene expression profiles induced by small molecules to profiles induced by gene knock-downs [26]. The 1,5-diaryl-3-oxo-1,4-pentadienyl compound F6 (NSC632839) (Figure 1) and curcumin are both present in the L1000 database. Curcumin induces a disparate response including p53 signaling, G-protein signaling, and zinc finger transcription factors (Table 2). By contrast, F6 induces a response characteristic of proteasome inhibition (Table 2), where the three top hits were proteasome subunits. b-AP15 and VLX1570 have previously been shown to induce gene expression profiles characteristic of proteasome inhibition [5,27].

### 3.2. VLX1570 Induces the Loss of Viability of Acute Myelocytic Leukemia (AML) Cells

Dienone compounds have been shown to be cytotoxic to a number of different cancer types, including multiple myeloma, colon cancer, melanoma, and acute lymphocytic leukemia [2]. VLX1570 (Figure 1) was advanced into a Phase 1 clinical trial. The trial was discontinued due to toxicity that was linked to the Chremophor-based formulation [14]. We were interested in examining the responses of AML cell lines and primary patient-derived AML cells to VLX1570 treatment. The properties of the AML cell lines used are presented in Table 3. Cells were exposed to various concentrations of VLX1570, and the IC_50_ values were determined using MTT assay (Table 3). The in vitro sensitivity of AML cell lines was found to be in the range of 200–300 nM. Two cell lines were examined in more detail, KG1a cells, harboring a mutation in the *TP53* gene, and MOLM-14 cells, reported to be *TP53*^wild-type^ (Table 3).

**Figure 2:**
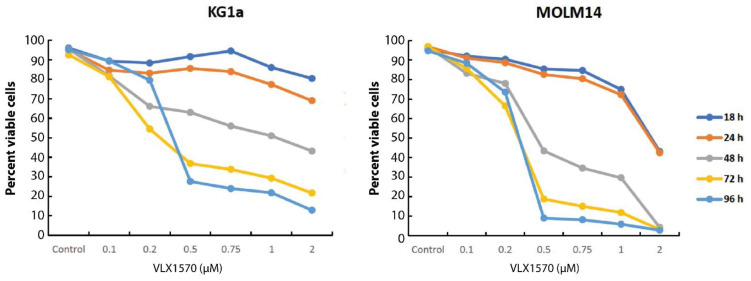
Time- and dose-dependent loss of viability of MOLM-14 and KG1a cells exposed to VLX1570. Cell viability was determined using acridine orange/propidium iodide staining.

KG1a cells showed a lower sensitivity to VLX1570 than MOLM-14 cells, both in MTT assays and in cell viability assays using acridine orange/propidium iodide staining [28] (Table 3 and Figure 2).

### 3.3. VLX1570 Induces Proteotoxic Stress and ER Stress in AML Cell Lines

**Figure 3:**
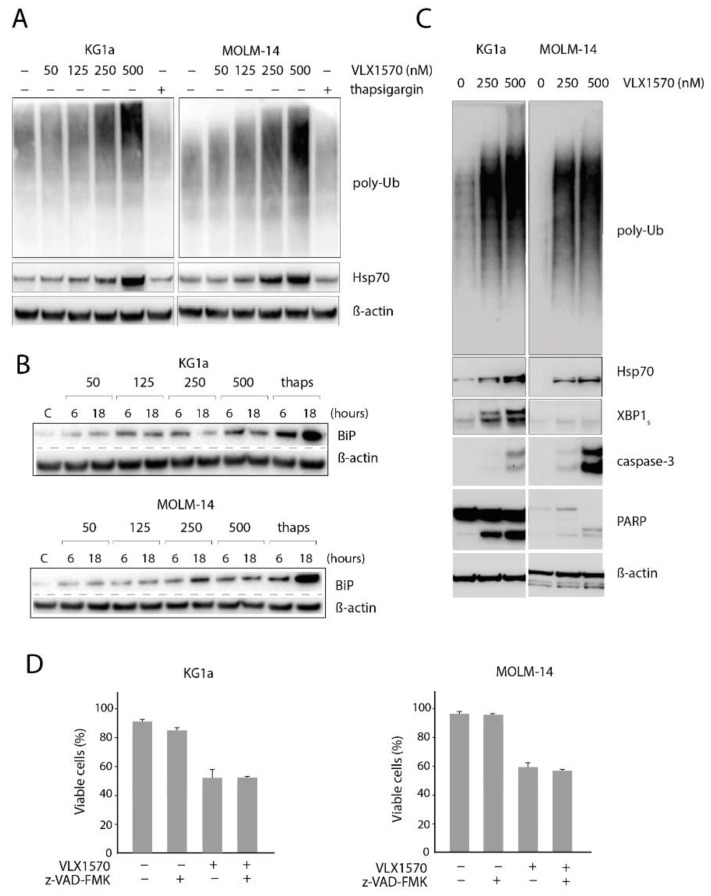
(**A**) Accumulation of polyubiquitinated proteins and increased in Hsp70 in KG1a and MOLM14 cells exposed to VLX1570; (**B**) time- and dose-dependent increased in Grp78/BiP in acute myeloid leukemia (AML) cells exposed to VLX1570; thaps: thapsigargin; (**C**) VLX1570 induction of proteotoxic stress and ER stress; (**D**) the loss of cell viability is not affected by the pan-caspase inhibitor z-VAD-fmk (10 μM). Cells were exposed to 0.35 μM VLX1570 for 48 h, and cell viability was determined by acridine orange/propidium iodide staining. The barplots are the average of three replicates of a single experiment. Error bars represent one standard deviation. The experiment was repeated with similar results.

Consistent with the analyses of gene expression profiles indicating the induction of proteasome inhibition by compounds containing 1,5-diaryl-3-oxo-1,4-pentadienyl pharmacophores, VLX1570 induced a dose-dependent increase in high-molecular-weight polyubiquitinated proteins in MOLM-14 and KG1a cells (Figure 3A). The increased levels of misfolded proteins are generally paralleled by the induction of molecular chaperones, and we indeed found that VLX1570 increased the expression of the inducible form of Hsp70 (HSP70B’) (Figure 3A). The increase in polyubiquitinated proteins and Hsp70 levels that occurred in the range of VLX1570 concentrations (250–500 nM) was concomitant with a dose-dependent reduction in cell viability, indicating a clear link between proteotoxicity and viability.

Proteasome inhibition is associated with the induction of ER stress [34], characterized by the induction of the ER chaperone Grp78/BiP, phosphorylation of eIF2α, and splicing of XBP1 mRNA [35]. We observed a dose- and time-dependent increase in Grp78/BiP in AML cells at pharmacologically relevant concentrations of VLX1570 (Figure 3B). The response of eIF2α was more variable. MOLM-14 cells expressed barely detectable levels of eIF2α, whereas KG1a cells showed constitutive eIF2α phosphorylation even under control conditions, making the interpretation difficult (Supplementary Figure S1). The increased expression of the spliced protein product of XBP1 mRNA (XBP1_s_) was strongly induced in KG1a cells but only weakly induced in MOLM-14 cells (Figure 3C). Taken together, our data suggest that VLX1570 causes a disruption in ER-mediated folding, as indicated by increases in BiP levels; however, the variations in other components of the ER stress are cell line dependent.

ER stress is associated with the induction of apoptosis [36]. The cleavage of PARP was observed in KG1a cells exposed to VLX1570 (Figure 3C), consistent with the strong induction of ER stress. Activation of caspase-3 was observed in both cell lines with a stronger activation observed in MOLM-14 cells. PARP was weakly expressed in MOLM-14 cells, but the complete cleavage was observed at 500 nM VLX1570.

Recent reports have linked apoptosis to cell death induction by VLX1570, either due to induction of ER stress [16] or depletion of anamorsin/CIAPIN1 [9]. Although our results support the view of induction of apoptosis by VLX1570, apoptosis is not necessarily required for cell death induction. We therefore examined whether caspase activity is essential for the loss of cell viability following the exposure to VLX1570. The pancaspase inhibitor z-VAD-FMK has previously been shown to inhibit apoptosis and PARP cleavage in cells and cell lysates, respectively [37]. The pretreatment of the cells with 10 µM z-VAD-FMK had no effect on VLX1570-induced AML cell death (Figure 3D), indicating that apoptosis is not a prerequisite for VLX1570-induced cell death.

### 3.4. VLX1570 Induces Glutathione Depletion and Formation of High-Molecular-Weight Protein Complexes

**Figure 4:**
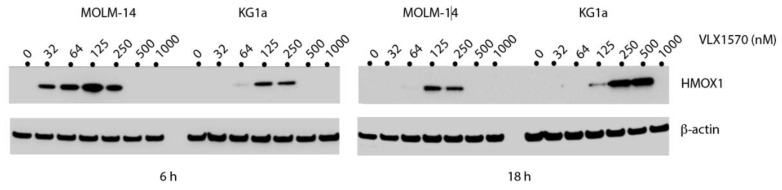
Induction of HMOX1 expression by VLX1570 in MOLM-14 and KG1a cells. Cells were exposed to the indicated concentrations of VLX1570 for 6 or 18 h and extracts processed for immunoblotting.

Dienone compounds are known to elicit oxidative stress [2,21]. HMOX1 (heme oxygenase-1) is an Nrf2 target gene previously shown to be induced by b-AP15 [38]. We found that HMOX1 was induced in MOLM-14 cells at 32 nM VLX1570 and at 64 nM VLX1570 in KG1a cells (Figure 4). HMOX1 was not, however, induced at high concentrations of VLX1570, consistent with previous observations of inhibition of elongation of translation at high concentrations of this compound [39]. We conclude that the Nrf2 target HMOX1 is induced at lower concentrations of VLX1570 in the more sensitive MOLM-14 cell line.

**Figure 5:**
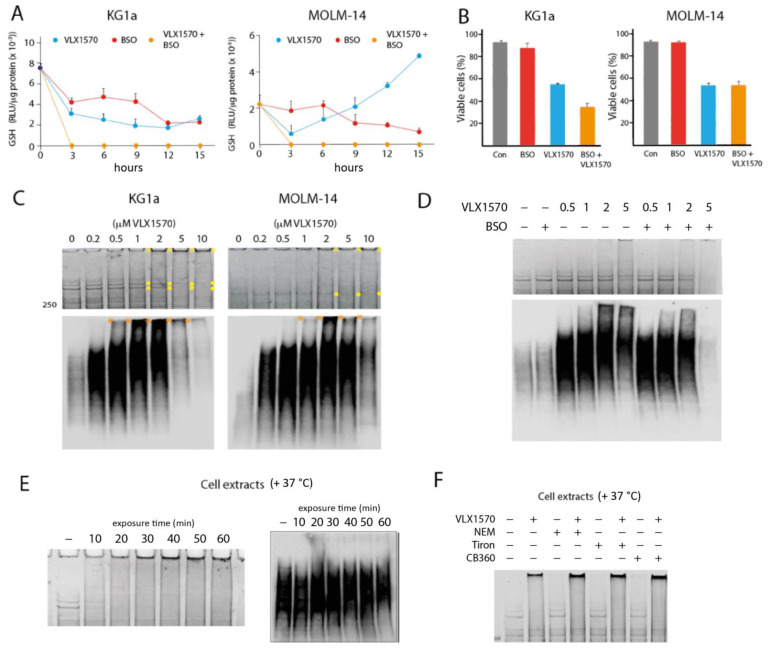
Depletion of reduced glutathione (GSH) and induction of high-molecular-weight complexes in AML cells. (**A**) GSH levels in AML cells after different times of exposure to 0.35 μM VLX1570 and/or 10 μM BSO. Means and SDs from one representative experiment are shown; (**B**) fraction viable cells determined by the AO/PI method after treatment with VLX1570 and/or BSO. Means and SDs from one representative experiment are shown; (**C**) (top) high molecular weight (HMW) region of Coomassie BB-stained SDS-PAGE gels loaded with extracts from cells exposed for 4 h to different concentrations of VLX1570, (bottom) immunoblots of extracts from VXL1570-exposed cells probed with an antibody to K48-linked polyubiquitin, note the distribution of high molecular weight bands marked with yellow and orange dots; (**D**) effect of 10 μM BSO on the formation of HMW complexes and loss of HMW proteins in KG1a cells exposed to 0.5, 1, 2, and 5 μM VLX1570; (**E**) KG1a cell extracts were exposed to 1 μM VLX1570 for increasing times at 37 °C and analyzed by SDS-PAGE; (left): gel stained with Coomassie blue, (right): immunoblot probed with an antibody to K48-linked polyubiquitin; and (**F**) effect of pretreatment of cell extracts with 20 μM NEM, 100 μM Tiron, or the Michael-acceptor-containing compound CB360 (30 μM) [40] on the formation of HMW complexes. Plots (**A**,**B**) show the average of three replicates, and error bars represent one standard deviation.

We hypothesized that the strong induction of HMOX1 by low concentrations of VLX1570 could be caused by the depletion of reduced glutathione (GSH), resulting from increased cellular oxidative and electrophilic stress. AML cells have been reported to be particularly sensitive to glutathione depletion by electrophilic compounds [17]. The levels of GSH decreased in a time- and dose-dependent manner following the exposure of KG1a and MOLM-14 cells to VLX1570 (Figure 5A). The response to VLX1570 differed between the two cell lines. In MOLM-14 cells, which are more sensitive to VLX1570, GSH levels recovered after an initial decrease and were higher than the levels of untreated cells after 12 h. By contrast, in KG1a cells, GSH levels did not recover within 15 h of treatment (Figure 5A). Interestingly, the stronger reduction of GSH in KG1a cells did not translate to a lower sensitivity of these cells to VLX1570 (Figure 2 and Table 3). To examine whether the levels of GSH decreases observed here are cytotoxic to AML cells, we used BSO (buthionine sulfoximine), an inhibitor of glutathione synthesis. BSO treatment resulted in decreases in GSH levels in both cell lines (Figure 5A) but did not affect the viability of either cell line (Figure 5B). These results suggest that the moderate degrees of GSH depletion observed using IC_50_ concentrations of VLX1570 are not related to the mechanism of cytotoxicity. The combination of BSO and VLX1570 reduced GSH to background levels in both cell lines (Figure 5A). Whereas the viability of KG1a cells was lower in cells exposed to both VLX1570 and BSO compared to VLX1570 alone, BSO did not affect the sensitivity of MOLM-14 cells to VLX1570 (Figure 5B). This discrepancy might reflect the higher baseline GSH levels found in KG1a (Figure 5A), which confers some resistance by the covalent inactivation of VLX1570. Nonetheless, these results are not consistent with the hypothesis that glutathione depletion plays an essential role in the mechanism of cytotoxicity of VLX1570 on AML cells.

Michael acceptor compounds have been reported to induce the formation of high molecular weight (HMW) protein aggregates in exposed cells [8,9,38]. The formation of such protein aggregates has been suggested to be the mechanism of cytotoxicity of VLX1570 [9]. We indeed observed the formation of protein material that did not enter SDS-polyacrylamide gels and material smearing from the wells using KG1a cells exposed to VLX1570 (Figure 5C). Bands in the high-molecular-weight region, in particular two bands of ~380 and 440 kDa (indicated with yellow circles in Figure 5C), decreased in intensity in parallel. The formation of HMW material was less pronounced in MOLM-14 cells, but smearing could be observed at >2 μM VLX1570 (Figure 5C). Polyubiquitinated proteins appeared to be more susceptible to complex formation, and HMW polyubiquitinated proteins were observed in KG1a cells exposed to 0.5–1 μM VLX1570 and in MOLM-14 cells at >2 μM (Figure 5C). To examine whether the appearance of HMW complexes could be a consequence of GSH depletion, we examined the effect of the BSO on KG1a cells (Figure 5D). BSO did not increase the formation of HMW material remaining in the wells or smearing on SDS-PAGE cells in cells exposed to VLX1570 (Figure 5D). Treatment with 5 μM VLX1570 in combination with BSO resulted in the loss of high-molecular-weight proteins (Figure 5D). Similar results were obtained when analyzing polyubiquitinated proteins (Figure 5D).

We next examined whether the HMW complex formation occurs in cell extracts exposed to VLX1570. As shown in Figure 5E, increases in proteins not entering the gel and smearing in the HMW region, as well as the loss of the 380/440 kDa bands, occur after the exposure of KG1a cell extracts to 10 μM VLX1570 for 10–60 min. Surprisingly, polyubiquitinated proteins present in cell extracts were unaffected by the exposure to VXL1570 (Figure 5E). We finally examined whether the treatment of cell extracts with N-ethylmalemide (NEM), the antioxidant Tiron, and the Michael acceptor compound CB360 [40] induced HMW complexes or whether pretreatment prior to the addition of VLX1570 affected the formation of such complexes. We found no effects of these compounds on the formation of HMW complexes (Figure 5F).

### 3.5. VLX1570 Inhibits Growth of AML Cells in Zebrafish Embryos

**Figure 6:**
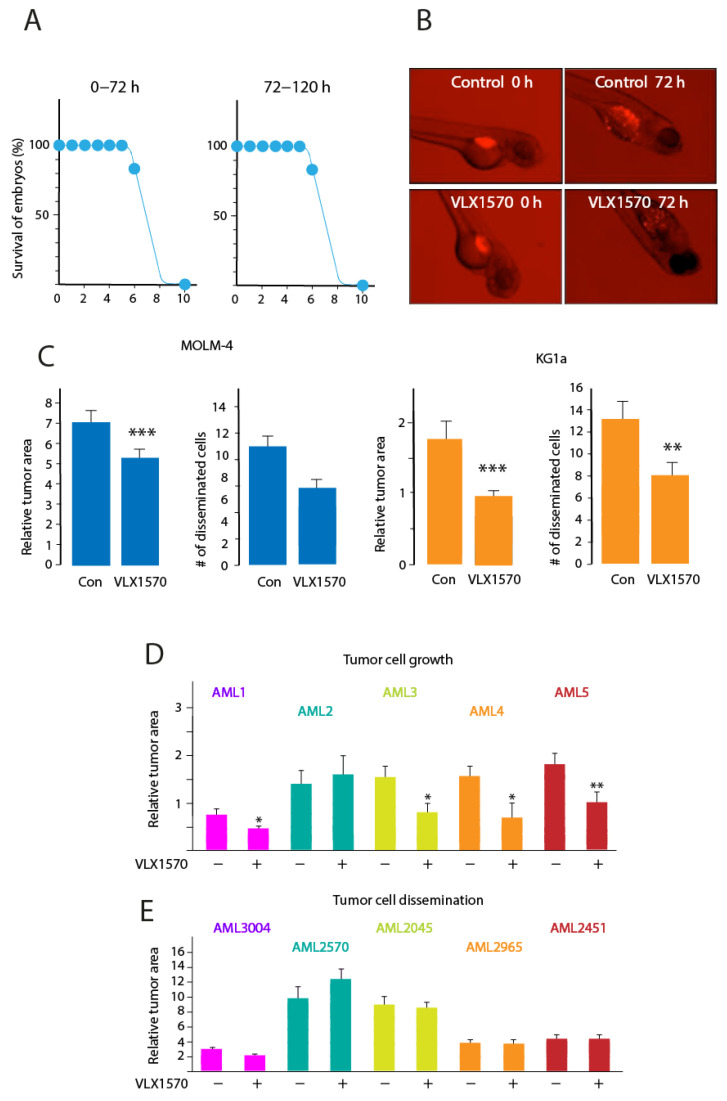
Activity of VLX1570 in the zebrafish embryo model; (**A**) survival of zebrafish embryos exposed to the indicated concentrations of VLX1570 for 0–72 or 72–120 h; (**B**) examples of results obtained using labeled AML cells injected into zebrafish embryos followed by treatment with VLX1570; (**C**) effect of 1 µM VLX1570 on the growth and dissemination of injected AML cell lines in zebrafish embryos (student’s *t* test, ** *p* ≤ 0.01, *** *p* ≤ 0.001); and (**D**,**E**) effect of 1 µM VLX1570 on the growth and dissemination of injected primary AML cells in zebrafish embryos (student’s *t* test, * *p* ≤ 0.05, ** *p* ≤ 0.01).

A key issue with this class of compounds is their solubility. The toxicity of VLX1570 is associated with the polyethylene glycol/polyoxyethylated castor oil/polysorbate 80 formulation used [14]. In order to examine the effect of VLX1570 on AML cells in an in vivo setting, we used a zebrafish embryo model where the compound could be added to the water. Zebrafish has been advocated as a suitable model for the assessment of toxicity and evaluation of in vivo activity of anticancer compounds [19,41]. VLX1570 did not affect embryonal development when added during the first three days of development or during day 3–5 at concentrations up to 5 μM (Figure 6A). To examine the effects on tumor cells, embryos were injected with dye-labeled MOLM-14 or KG1a cells after eight hours of development. VLX1570 (1 μM) was then added, and the effects were evaluated after an additional 72 h incubation. Both tumor cell growth and spread to distant sites (a model for metastatic dissemination [18]) were significantly inhibited for KG1a and MOLM-14 cells (Figure 6B,C).

We next examined five clinical cases of AML. The genetic make-up of these cells varied as shown in Table 4. The mutation of p53 was not detected in any of these cases. Cells were labeled and injected into embryos and labeled cells at the injection site and in the tail were assessed. Decreased tumor cell growth/viability was observed for 4 out of 5 samples (Figure 6D). Two patient samples displayed complex karyotypes (AML2 and AML5). Decreased growth was observed for one of these samples (Figure 6D). VLX1570 did not, in contrast, have any significant effect on the dissemination of patient-derived AML cells (Figure 6E).

## 4. Discussion

Compounds containing the 1,5-diaryl-3-oxo-1,4-pentadienyl pharmacophore have attracted the interest of a number of laboratories due to their documented tumor cell selectivity [2,3,42]. These compounds are flagged by PAINS filters [43], and whether they can trigger a defined response is debatable. The natural product curcumin has a structure related to 1,5-diaryl-3-oxo-1,4-pentadienyl compounds and has been widely studied as a potential anticancer agent [42,44]. Strong arguments have been raised arguing that the general reactivity, poor bioavailability, and poor pharmacokinetic properties of curcumin disqualifies the compound from being a drug candidate [45,46]. An analysis of the Broad Institute LINCS database showed that curcumin and F6 induce distinct transcriptional responses and that the F6 response is characteristic of proteasome inhibition. Previous analyses of the transcriptional response to VLX1570 in colon cancer cells [27] and AML cells [16] are similar to that of proteasome inhibitors, showing the induction of chaperones, heme oxygenase-1, and immediate early-response genes. The compounds P1 and P2, both containing the 1,5-diaryl-3-oxo-1,4-pentadienyl pharmacophore, were also found to induce a gene expression profile characteristic of proteasome inhibition [47]. Previous studies using reporter proteins have shown a strong association between the ability of b-AP15 to inhibit proteasome processing and to induce cell death (see [2]), arguing that 1,5-diaryl-3-oxo-1,4-pentadienyl compounds do induce a dominant pharmacological response despite their PAINS properties.

Consistent with a recent report [16], we found that VLX1570 induces the accumulation of polyubiquitinated proteins and of Hsp70 in AML cells at the concentration range where the loss of cell viability is observed. Similar results have been reported using other types of tumor cells [39,48]. We also found a dose- and time-dependent induction of the ER chaperone Grp78/BiP in MOLM-14 and KG1a AML cells, indicating the induction of ER stress. In the VLX1570-sensitive MOLM-14 cell line, we only observed a weak induction of the spliced form of XBP1. There was no consistent pattern in the induction of ER stress markers, activation of caspase-3, and cleavage of PARP in the two cell lines. This result could be interpreted as suggesting that ER stress is one of several parallel mechanisms that lead to apoptosis, and it also raises the question of whether apoptosis-independent cell death mechanisms are activated. We indeed found that the pancaspase inhibitor z-VAD-fmk does not impede VLX1570-mediated cell death, suggesting that ER stress-induced apoptosis is not of critical importance for the cytotoxic activity of VLX1570 on AML cells. The underlying mechanism resulting in caspase-independent cell death is unclear but may be related to the induction of strong proteotoxic stress resulting from the ineffective formation of cytoprotective aggresomes [49]. Cell death occurring independently of apoptosis is not consistent with a recently proposed mechanism of cell death involving the depletion of the apoptosis inhibitor anamorsin/CIAPIN1 [9] but is interesting with regard to the potential antineoplastic activity of this class of compounds to tumor cells showing defects in apoptotic pathways.

We found that VLX1570 concentrations in the cytotoxic range reduced the levels of glutathione in AML cells (Figure 5). The possibility that the reduction of GSH contributes to the cytotoxicity of the compound was considered. We were surprised to find that the inhibition of glutathione synthesis using BSO did not affect AML cell viability. In the VLX1570-sensitive MOLM-14 cell line, GSH levels recovered within 12 h. Since >80% viability was observed 24 h after the exposure of MOLM-14 cells to 0.5 μM VLX1570 (Figure 2), it appears unlikely that glutathione depletion is mechanistically involved in eliciting cytotoxicity. Furthermore, BSO did not further reduce the viability of MOLM-14 cells exposed to VLX1570, despite the reductions of GSH to undetectable levels. These findings are not consistent with those previously reported [17], but the differences could be explained by the use of the primary AML cell in that study.

It was recently suggested that the mechanism of cytotoxicity of VLX1570, and presumably the entire class of related compounds, is associated with the formation of HMW protein aggregates [9]. Although it is known that protein aggregation can have toxic effects regardless of the identity of the aggregating protein [50], this hypothesis is difficult to reconcile with the tumor cell selectivity of 1,5-diaryl-3-oxo-1,4-pentadienyl compounds. We here confirm that VLX1570 does indeed induce HMW protein material, both in exposed cells and using cell extracts. The formation of this material did, however, not occur at concentrations that resulted in the loss of cell viability. The formation of HMW protein was observed at 2 μM in MOLM-14 cells, ~10-fold the IC_50_, and ~50-fold higher than the concentration found to induce HMOX1. Surprisingly, treatment with BSO did not further increase the formation of HMW material. A previous study demonstrated that curcumin induces the dimerization of the crosslinks cystic fibrosis transmembrane conductance regulator (CFTR) [51]. This phenomenon also occurred using a CFTR protein lacking cysteine residues, and the authors speculated that dimerization was caused by an oxidative process [51]. We found that HMW aggregates were formed in the presence of the antioxidant Tiron and were not affected by pretreatment with the cysteine-reactive compound NEM. We also found that the mono-enone compound CB360 did not induce these aggregates or prevent their formation. Polyubiquitinated proteins present in living cells were found to be more prone to the formation of HMW material than the bulk of proteins (Figure 5C). A possible explanation for this phenomenon would be that misfolded proteasome substrates are more accessible to adduct formation by VLX1570 compared to native proteins. However, and surprisingly, polyubiquitinated proteins present in cell extracts were less sensitive than bulk proteins compared to the VLX1570-induced formation of HMW material. We conclude that, although the mechanism of the formation of HMW aggregates by VLX1570 is unclear, the observation that VLX1570 concentrations are considerably higher than those resulting in cytotoxicity does not support the notion that this phenomenon is important for the mechanism of action of the compound.

*TP53* mutations occur in only 8% of de novo AML cases, one of the lowest frequency rates among human malignancies (TCGA research network: https://www.cancer.gov/tcga, accessed on 7 June 2021). However, *TP53* mutations are associated with dismal survival rates and thereby represent important factors influencing the prognosis of patients with AML [12]. The reactivation of mutant forms of p53 by small molecules has been an active field of investigation [52]. The finding that the dienone compound HO-3867 (Figure 1) reactivates mutant p53 [53] is therefore of interest with regard to AML treatment. Our finding that *TP53*^wt^ MOLM-14 cells are more sensitive than p53^mut^ KG1a cells is not consistent with a model where p53 reactivation is a major mode of action of VLX1570. The five primary AML cases that were used were all p53^wt^ and found to be sensitive to VLX1570 in the zebrafish model, again showing that p53 reactivation is not required for the cytotoxic activity of VLX1570 on AML cells. We note that the other dienone compounds (Supplementary Figure S2) did not show the differences in the IC_50_ between p53 mutant and wild-type tumor cell lines in the NCI_60_ cell line panel, raising the possibility that reactivation of p53 is not a general property of this class of compounds. The notion that the VLX1570 sensitivity of AML cells does not correlate to mutational status of *TP53* is also supported by the results of Kurozumi et al. [16].

AML is a potentially interesting field of application for this class of compounds as AML cells are sensitive to UPS perturbations [54,55] and to glutathione depletion by electrophiles [17]. Our results show that AML cell lines and primary patient cells are sensitive to VLX1570. The finding that the ability of VLX1570 to induce cell death is apoptosis independent is attractive considering the treatment resistance of relapsed AML. Another interesting property of this class of compounds is the limited development of resistance during the long-term culture of cells in the presence of drug [56]. These properties render this class of compounds to be candidates for treatment of AML, a serious disease with a clear medical need. A problem encountered with VLX1570 and various analogs is the limited compound solubility. The formulation used for VLX1570 was found to contribute to systemic toxicity [14]. We here used a zebrafish embryo xenograft model that does not require the formulation of VLX1570 since the compound could be added to the water. The growth of four out of five patient AML samples at the injection site was reduced using a VLX1570 concentration that was >5-fold lower than the concentration that caused developmental toxicity (for a discussion of the zebrafish embryo xenograft, see [41]). By contrast, dissemination of AML cells in the embryos was not reduced, possibly due to disseminated cells being quiescent after dissemination and not sensitive to VLX1570 [56]. These findings provide a proof of principle for the neoplastic effect of VLX1570 in AML. However, more soluble analogs or improved formulations need to be developed in order to expand the applicability of this interesting compound.

## Figures and Tables

**Table 1:** Properties of compounds containing the 1,5-diaryl-3-oxo-1,4-pentadienyl pharmacophore and of the natural product curcumin.

Compound	Described Target(s)	Calculated Solubility ^a^	PAINS ^b^	LogP
b-AP15	USP14/UCHL5	−4.43	yes	2.08
VLX1570	USP14/UCHL5	−5.14	yes	2.91
RA-9	USP14/UCHL5	−3.95	yes	1.75
RA-190	Rpn13/UCHL5	−8.22	yes	5.49
F6 (NSC632839)	Deubiquitinases	−5.15	yes	3.60
EF24	NFkB, MAPK, HIF-1α	−4.86	yes	3.62
Curcumin	Multiple targets	−3.94	no	3.03

^a^ Calculated solubility, LogS (ESOL). ^b^ Pan-assay interference compounds.

**Table 2:** Top gene knockdown expression profiles showing the greatest connectivity with F6 or curcumin.

	NSC-632839		Curcumin	
Rank	Gene	Description	Gene	Description
1	*PSMA1*	Proteasome subunit	*TP53RK*	Bud32 family
2	*PSMD1*	Proteasome subunit	*CD46*	CD molecules
3	*PSMA3*	Proteasome subunit	*ZNF418*	Zinc finger
4	*PSMD3*	Proteasome subunit	*KLF2*	Kruppel-like transcription factor
5	*PSMB2*	Proteasome subunit	*RGS18*	Regulator of G-protein signaling
6	*VCP*	AAA ATPase	*PI3KCB*	Phosphatidylinositol kinase
7	*PSMB5*	Proteasome subunit	*ZNF350*	Zinc finger
8	*HSPA5*	Heat Shock Protein	*ZNF67*	Zinc finger
9	*CEBPG*	Basic Leucine Zipper	*ATP6V0A1*	V-ATPase
10	*RYK*	Type XV RTK	*ACACA*	Carboxylase

**Table 3:** AML cell lines used in the present study.

Cell Line	Cell Line Properties	VLX1570 IC_50_ (nM)
KG1a	Complex karyotype [11], p53mut [29]	307 ± 31
MOLM-14	49,<2n>,XY,+6,+8, ins(11;9)(q23;p22p23), del(14)(q13.2q31.3) fusion gene MLL-AF9 [30], p53wt [30,31]	204 ± 73
HNT34	46,XX, t(3;3)(q21;q26), t(9;22)(q34;q11),20q− [32]	273 ± 61
Kasumi-1	Human hypodiploid karyotype—45,<2n>,X,−Y,−9,−13,−16, +3mar, t(8;21)(q22;q22), der(9)t(9;?)(p22;?), der(15)t(?9;15)((?q11;?p11) [33]	260 ± 27

**Table 4:** Primary AML cells used in the present study.

Patient	Age	Karyotype	Genotype
AML1	80	46,XX	NPM1^pos^; FLT3^neg^
AML2	65	46,XY, t(4;11)(q35;q23) [15] 46,XY [5]	
AML3	72	46,XX	NPM1^pos^; FLT3-ITD^pos^
AML4	31	46,XX	CEBPA^pos^; WT1^mut^; CSF3R^mut^; KIT^mut^
AML5	48	46,XY, inv16(p13q22) [3] 47,XY,+8, inv16(p13q22) [16] 46,XY [2]	

## Data Availability

The data presented in this study are available in article or Supplementary Files.

## Supplementary Materials

The following are available online at https://www.mdpi.com/article/10.3390/biom11091339/s1, Figure S1: Analysis of markers of ER stress and apoptosis in AML cells exposed to VLX1570, Figure S2: The sensitivity of cancer cell lines to dienone compounds is not affected by p53 mutational status.
