# Supplementary material for: Sensitivity of Acute Myelocytic Leukemia Cells to the Dienone Compound VLX1570 Is Associated with Inhibition of the Ubiquitin-Proteasome System
Source: Biomolecules. 2021 Sep 10;11(9):1339. doi: 10.3390/biom11091339 (PMC8470745; doi:10.3390/biom11091339)
Supplement: Supplementary file 1 [file biomolecules-11-01339-s001.zip › biomolecules-1308072-supplementary.pdf]

Supplementary Materials

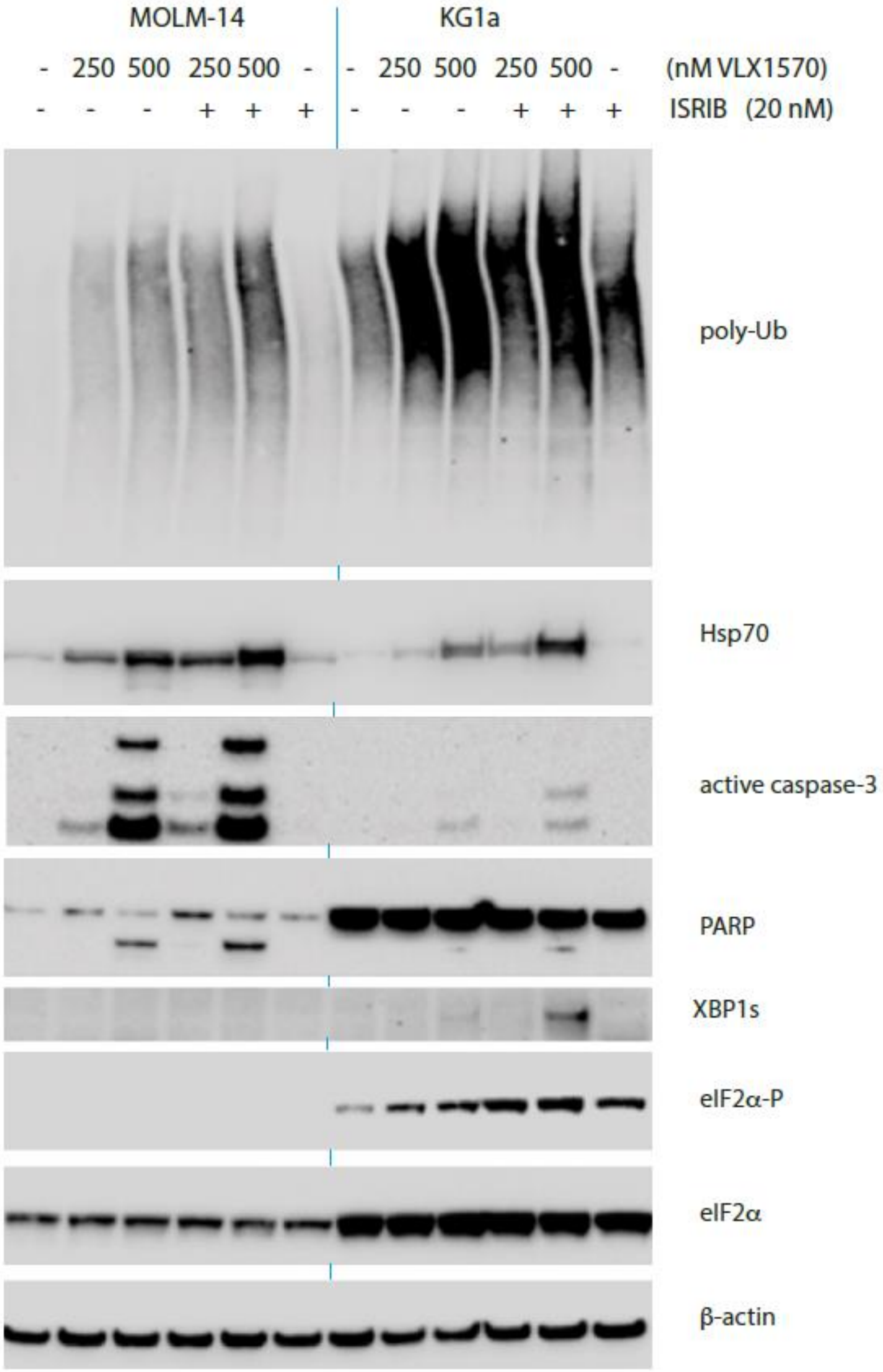

Figure S1. Analysis of markers of ER stress and apoptosis in AML cells exposed to VLX1570. MOLM-14 cells express eIF2α and caspase-3 at different levels. Cells were exposed to ISRIB (integrated stress response inhibitor) as indicated. ISRIB is known to reverse the effects of eIF2α phosphorylation due to the dimerization of eIF2B (Sidrauski et al. (2015), PMID 25719440). Note the increase in Hsp70 in cells exposed to VLX1570 and ISRIB, suggesting that eIF2α phosphorylation restrict proteotoxic stress by inhibition of translation. In addition, note the slight increases in active caspase-3 and cleaved PARP in cells exposed to ISRIB.

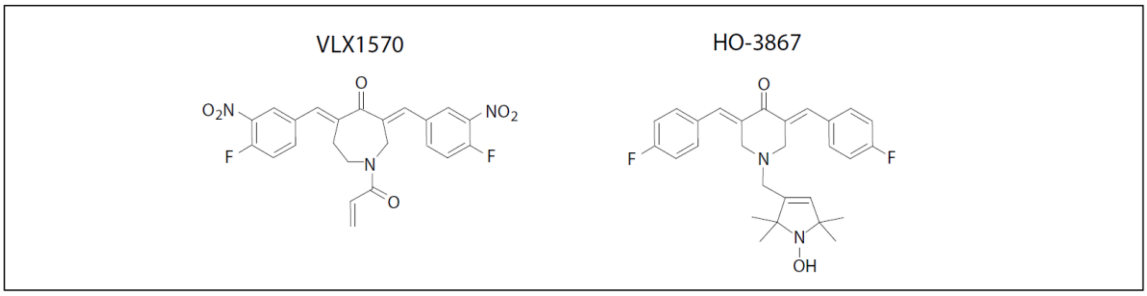

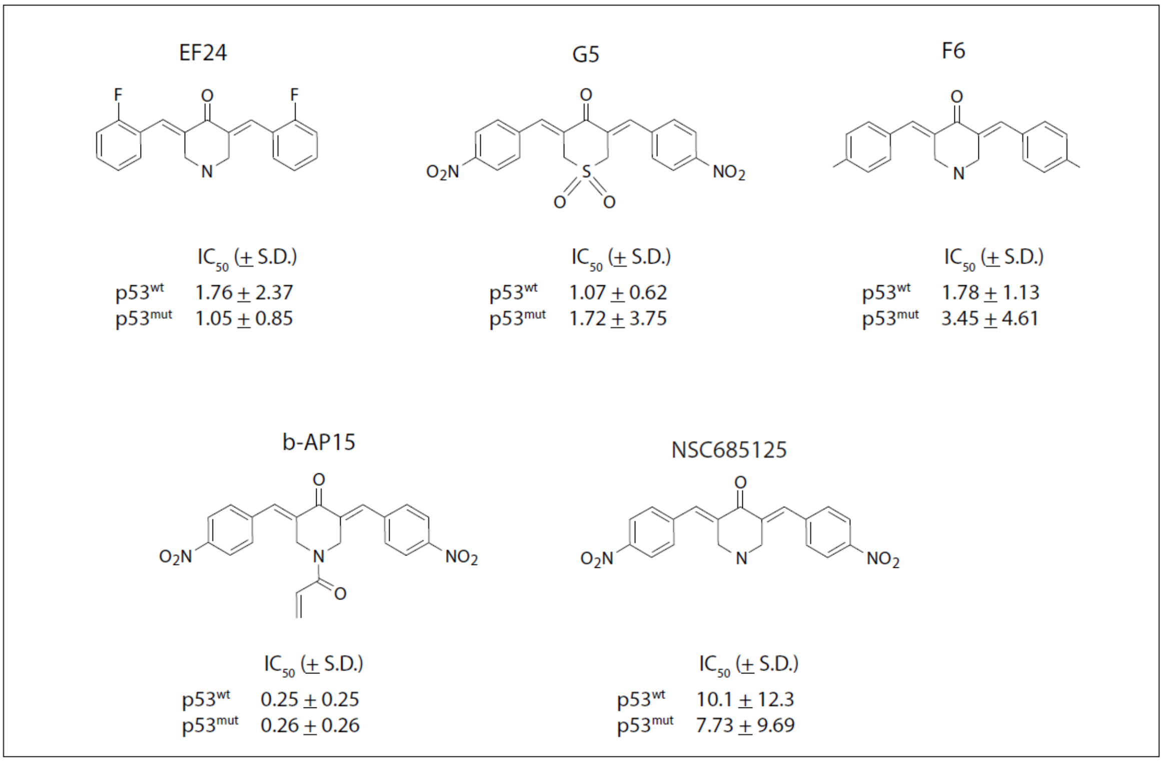

Figure S2. The structures of VLX1570 and HO-3867 are shown in the top panel. Both compounds are dienones with electron-drawing groups on the flanking aryl groups. The structures of a number of other dienones that have been tested in the NCI developmental therapeutic program are shown together with mean GI50 values for p53wt and p53 mutant tumor cell lines. No trend for increased sensitivity of dienone compounds of cells with mutant p53 can be discerned. GI50 values are from the NCI developmental therapeutic program /dtp.cancer.gov.

p53 wild-type cell lines: SR, A549, NCI-H460, HCT116, SK-MEL-5, UACC-257, A498, ACHN, CAKI-1, UO-31, MCF7.

p53 mutant cell lines: CCRF-CEM, HOP-92, NCI-H23, NCI-H322M, HCC-2998, HT29, SW-620, SF-268, SF-539, SNB-75, U251, M14, SK-MEL-28, OVCAR-3, RXF393, SN12C, TK-10, PC-3, MDA-MB-231, HS578T, BT-549, T47D.

(We have used the evaluation of the p53 mutation status in the NCI60 panel published by Berglind et al. (2008))

HO-3867 (Selvendiran et al. (2010)), EF24 (Adams et al. (2005)), G5, F6 (Aleo et al. (2006))
